# Mesenchymal to Epithelial Transition Induced by Reprogramming Factors Attenuates the Malignancy of Cancer Cells

**DOI:** 10.1371/journal.pone.0156904

**Published:** 2016-06-03

**Authors:** Mikiro Takaishi, Masahito Tarutani, Junji Takeda, Shigetoshi Sano

**Affiliations:** 1 Department of Dermatology, Kochi Medical School, Kochi University, Nankoku, Japan; 2 Department of Social and Environmental Medicine, Graduate School of Medicine, Osaka University, Suita, Japan; University of North Carolina School of Medicine, UNITED STATES

## Abstract

Epithelial to mesenchymal transition (EMT) is a biological process of metastatic cancer. However, an effective anticancer therapy that directly targets the EMT program has not yet been discovered. Recent studies have indicated that mesenchymal to epithelial transition (MET), the reverse phenomenon of EMT, is observed in fibroblasts during the generation of induced pluripotent stem cells. In the present study, we investigated the effects of reprogramming factors (RFs) on squamous cell carcinoma (SCC) cells. RFs-introduced cancer cells (RICs) demonstrated the enhanced epithelial characteristics in morphology with altered expression of mRNA and microRNAs. The motility and invasive activities of RICs *in vitro* were significantly reduced. Furthermore, xenografts of RICs exhibited no lymph node metastasis, whereas metastasis was detected in parental SCC-inoculated mice. Thus, we concluded that RICs regained epithelial properties through MET and showed reduced cancer malignancy *in vitro* and *in vivo*. Therefore, the understanding of the MET process in cancer cells by introduction of RFs may lead to the designing of a novel anticancer strategy.

## Introduction

Cancer represents a life-threatening condition with increasing mortality when it metastasizes. Although the mechanisms of cancer metastasis are not yet fully understood, it is known that the acquisition of invasive properties is driven by epithelial to mesenchymal transition (EMT) [1, 2]. Cancer under EMT is characterized by a loss of intercellular adhesions, a gain of cell motility, and increased invasive activity. A major characteristic of EMT is the functional loss of the adherence junction protein, E-cadherin (CDH1), through multiple mechanisms, including gene mutation, hypermethylation, and repression of its promoter [1, 3, 4].

Repressive transcription factors for CDH1 include the (a) Snail family (SNAI1 and SNAI2) [5, 6], (b) Twist family (TWIST1 and TWIST2) [7, 8] and (c) ZEB family (ZEB1/dEF1 and ZEB2/SIP1) [9–11]. Those transcription factors can interact with the E-box element of the *CDH1* promoter, leading to the down-regulation of its expression. The over-expression of those EMT-inducing genes is frequently observed in the metastatic cancer cells [1, 3, 4]. On the contrary, the down-regulation of EMT-inducing gene expression restores *CDH1* expression and leads to the attenuation of cancer malignancy through a mechanism referred to as mesenchymal to epithelial transition (MET), the reverse program of EMT [1, 12–14].

MicroRNAs (miRNAs) are small non-coding RNAs that regulate their target genes expression at the post-transcriptional level and are known to play essential roles in different types of cancer. It has been reported that the EMT-inducing transcription factors, which suppress the *CDH1* expression, are negatively regulated by the miRNAs (miR-200 family, miR-203, and miR-205, etc.) [15–17].

During the generation of induced pluripotent stem cells (iPSCs) from murine fibroblasts by the introduction of four reprogramming factor genes, Oct3/4, Sox2, Klf4, and Myc, the cells went through MET [18, 19]. While iPSCs have been generated from the primary cells, recent studies have explored the possibilities of reprogramming the malignant cells, including leukemia, sarcoma, melanoma and other types of cancer cells to exhibit an iPSC-like state *in vitro* [20–25]. The reprogrammed malignant cells showed a pluripotent-like state with an altered differentiation program that led to the loss of tumorigenicity. However, it was uncertain whether cancer malignancy was attenuated through the MET-mediated mechanism with reprogramming factors.

Thus, the aim of the present study is to demonstrate that the SCC cells decrease malignant potential *in vitro* and *in vivo* through MET by the introduction of reprogramming factors without the pluripotent-like state. These findings are highly relevant for developing new and effective therapeutic strategies for cancer therapy.

## Materials and Methods

### Generation of iPS cells using piggyBac transposon system

Normal human epidermal keratinocytes (NHEKs) isolated from foreskins were obtained from Kurabo (Osaka, Japan) and were grown in Epilife II (Invitrogen) supplemented with Humedia-KG (Kurabo). pCMVmPBase and plasmids containing the piggyBac transposon carrying the reprogramming factors (POU5F1 (OCT3/4), SOX2, KLF4, cMYC, and LIN28) [26, 27] were transfected into the NHEKs with NEON transfection system (Invitrogen). The transfected NHEKs were immediately inoculated on feeder cells in Primate ES Cell Medium (ReproCell, Yokohama, Japan) supplemented with bFGF (4ng/ml), Y-27632 (10 μM), CHIR99021 (3 μM), PD0325901 (0.5 μM) and SB431542 (5 μM), all those reagents were from Wako pure chemical Industries (Osaka, Japan). The medium was changed to fresh one every other day. ES cell like colonies appeared on days 10–14, and were picked and expanded further on approximately day 21. To investigate whether the iPS cells derived from NHEKs has ES-like phenotype, the cells were stained with alkaline phosphatase substrate kit (Vector laboratories, Burlingame, CA), and with anti-Nanog antibody (Abcam, Cambridge, UK). The iPS cells were inoculated into testes of SCID mice (CLEA Japan, Tokyo, Japan) under anesthesia and the mice were euthanized to excise the testes at day 60 after the cell transplant. The testes were fixed with formalin and processed for paraffin section for histopathological examination.

### Cell lines

Human SCC cell lines, HOC313 and TSU [28, 29], were gifted by Dr. Kamata, Institute of Biomedical & Health Sciences, Hiroshima University, Japan. The OSC-19 cells and NCCIT cells were purchased from the Japanese Collection of Research Bioresources Cell Bank (Ibaraki, Japan). All the cell lines were maintained in Dulbecco's Modified Eagle Medium (DMEM; Thermo Fisher Scientific, Waltham, MA, USA) supplemented with 10% Fetal Bovine Serum (FBS) and antibiotics.

### Introduction of reprogramming factors

The PiggyBac transposon vector and the transposase-expression vector were co-transfected to the subconfluent SCC cells using Fugene 6 (Roche, Basel, Switzerland) reagent. Two days after the transfection, puromycin at final concentrations of 1~5 μg/ml was added to the cell culture medium for selection. The cells were maintained in DMEM supplemented with 10% FBS and antibiotics.

### Cell morphological assessment

The changes in cell morphology were evaluated by calculating the length to width ratio of each cell under a microscope using 20 cells per group.

### Quantitative real-time RT-PCR

The total RNAs were extracted using an RNAeasy kit (Qiagen, Venlo, the Netherlands) and were reverse-transcribed using random hexamers and Superscript III (Thermo Fisher Scientific). Each cDNA was subjected to quantitative PCR, using Power SYBR Green PCR Master Mix on Sequence Detection Systems 7300 (Thermo Fisher Scientific). Relative mRNA expression was normalized to the expression of hypoxanthine guanine phosphoribosyl transferase (HPRT). The primers used in this study have been listed in S1 Table.

### Detection of microRNAs

The total RNAs were prepared from the cultured cells using a miRNeasy mini kit (Qiagen). In accordance to the manufacturer’s instructions, each template cDNA was prepared using a miScript II RT kit (Qiagen) and the expression of microRNAs was assayed using a miScript SYBR Green PCR kit along with miScript Primer Assay (Qiagen). This was followed by the normalization of miRNA with SNORD61.

### MTS assay

A total of 96-well plates were considered for the study that contained 1000 cells each, which were seeded. One and two days later, the MTS reagent (Promega, Madison, WI, USA) was added to each well and incubated at 37°C for two hours. Subsequently, the absorbance values were also determined at 490 nm using a spectrophotometer.

### Immunofluorescent staining

Cells grown on chamber slides (Thermo Fisher Scientific) were fixed either with 4% paraformaldehyde in PBS solution at 4°C or 100% methanol at −20°C, and blocked with serum-free blocking solution (Dako, Glostrup, Denmark). Primary antibodies were incubated at 4°C overnight, followed by washing in PBS and incubation with secondary antibodies conjugated with Alexa Fluor 488 (Thermo Fisher Scientific). The nuclei were stained with 4’,6-diamidino-2-phenylindole, dihydrochloride (DAPI). The primary antibodies used have been listed in S2 Table. Fluorescence images were captured using a microscope with a charge-coupled device camera system (DP-71; Olympus, Tokyo, Japan).

### Immunohistochemistry

The paraffin-embedded sections obtained from the xenotransplants were deparaffinized and incubated in 10 mM citrate buffer (pH 6.0) for 10 min at 105°C. The sections were incubated with primary antibodies overnight at 4°C, followed by washing in PBS and incubation with the respective secondary antibodies (Dako). The signals were visualized using 3,3’-diaminobenzidine tetrahydrochloride (DAB). Nuclei were counterstained with hematoxylin. The primary antibodies used in this study are listed in S2 Table.

### Flowcytometry

Cell suspension was incubated with the anti-TRA-1-60 (Millipore), TRA-1-81 (Millipore), or mouse IgM (Bioledend) as isotype control for 30 min on ice and followed by incubation with Alexa fluor 488 conjugated anti-mouse IgM antibody. The cells were analyzed on an LSRFortessa flow cytometer (BD BioSciences) using FlowJo software (FlowJO, Ashland, OR).

### Electron microscopy

Fully confluent cells in 35 mm dishes were fixed with 2% glutaraldehyde in 0.1 M phosphate buffer (pH 7.3) and post-fixed with 1% osmium tetroxide, followed by 5% sucrose in 0.1 M phosphate buffer. Propylene oxide was used to dissolve the culture dishes to obtain the cell sheets. The ultra-thin sections were observed using a Hitachi H-7100 electron microscope (Hitachi High-Technologies, Tokyo, Japan).

### Western blot analysis

The cell lysates were prepared using radioimmunoprecipitation assay buffer (RIPA buffer; Sigma-Aldrich, St-Louis, MO, USA) or urea solution (9 M urea, 2% Triton-100, 5% 2-mercaptoethanol), separated on 4–15% gradient gels (Bio-Rad, Richmond, CA, USA) and blotted on polyvinyl difluoride (PVDF) membranes (Bio-Rad). An ECL Prime kit (GE Healthcare, Little Chalfont, UK) was used for signal detection. The antibodies used are listed in S2 Table. ImageJ (NIH) was used for quantification of the signals.

### *In vitro* wound healing assay

Fully confluent cells in six well-plates were treated with mitomycin C (10 μg/ml) for three hours, followed by scratch wounding using a yellow micropipette tip and washing with a pre-warmed medium. Wound healing by migrating cells was observed using a phase contrast microscopy and was photographed at the specified times. The distance of cell migration across the wound was measured over time.

### Cell invasion assay

*In vitro* cell invasion was evaluated using Boyden chambers in the presence of matrigel coating (Corning, Corning, NY, USA). A total of 25 × 10^3^ cells were inoculated into the upper chambers of 24-well plates and incubated for 24 hours. The cells attached to the membrane between the upper and lower chambers were stained with toluidine blue and counted under a microscope.

### Xenotransplantation of human SCC cells into the nu/nu mice

For orthotopic model, the cultured parental or the reprogramming factors introduced OSC-19 cells, which were suspended in PBS at a density of 1.0 × 10^7^ cells / ml. Under the isoflurane anesthesia, 2.0 × 10^5^ viable cells were inoculated into the lingual margin of female seven-week old BALB/c-nu/nu mice (Japan SLC, Hamamatsu, Japan). Body weight of the mice was measured three times per week to monitor their condition until experimental end point. The tongues and draining lymph nodes were dissected from the euthanized mice at day 21. The tongues were fixed with formalin and processed for histological analysis. The tumor areas in the tongue tissues were calculated under a microscope using the application software (DP2-BSW, Olympus). Total RNA was extracted from the cervical lymph nodes, and the gene expression of human cytokeratin 18 was assessed through quantitative real-time RT-PCR. For distant organ metastasis model, the parental OSC-19 cells and the RICs were suspend at a density of 1.0 × 10^6^ cells / ml and 2.0 × 10^5^ cells were transplanted via tail vein of female nu/nu mice. Day 47 after the cell transplant, the mice were euthanized and followed by necropsy. Cranial lobe of right lung was used for mRNA extraction and followed quantitative real-time RT-PCR to detect gene expression of human cytokeratin 18. Left lung, liver, kidney and spleen were fixed with formalin and processed for histological analysis. It was ensured that all the experiments involving mice strictly adhered to the institutional guidelines set for minimizing distress to animals. The study was approved by the Institutional Animal Care and Use Committee of the Kochi Medical School.

### Statistical Analysis

Statistical analysis was performed with Prism software (GraphPad, CA, USA) using an unpaired Student *t* test and Fisher’s exact test.

## Results

### Introduction of Reprogramming Factors (RFs) into SCC Cells Restores Epithelial Morphology but Does Not Provide the Pluripotency

**Fig 1:**
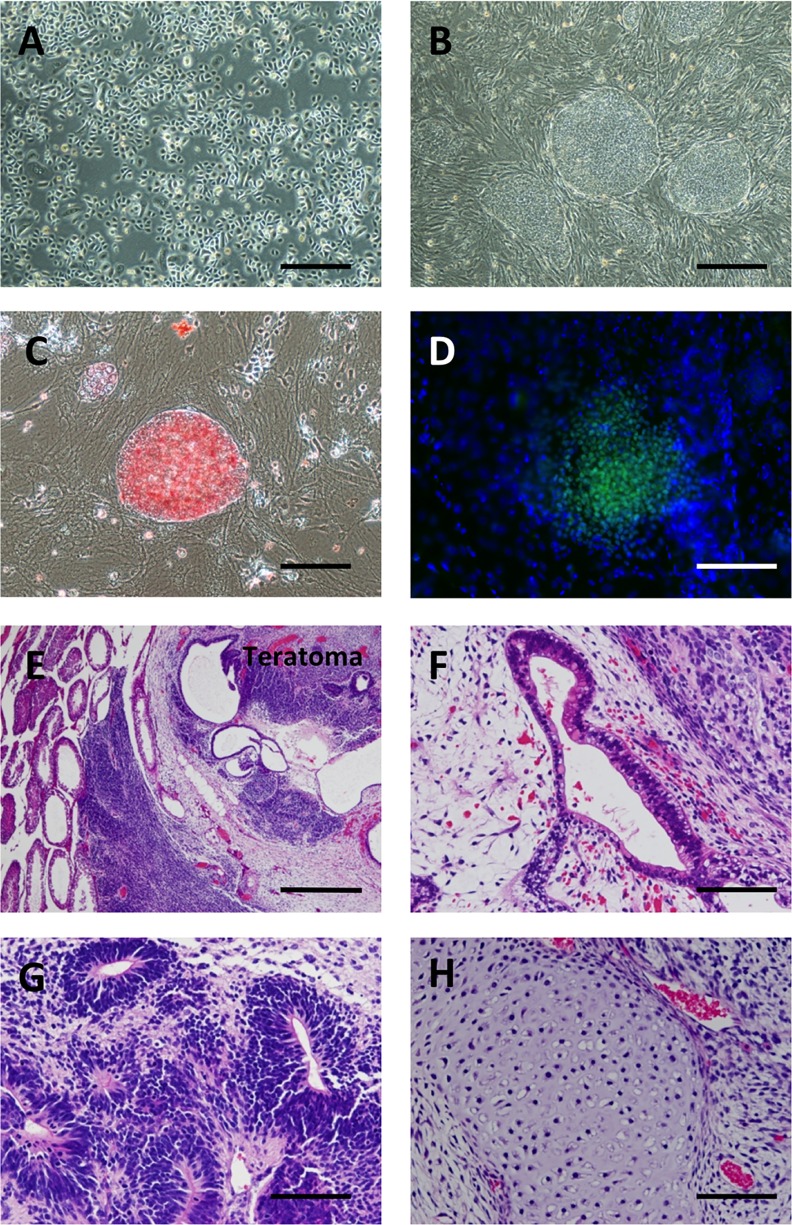
Generation of iPS cells from normal human epidermal keratinocytes. (A, B) Phase contrast image of normal human epidermal keratinocytes (NHEK) (A) and iPS cells generated from NHEK by introduction of the piggyBac transposon vectors (B). Bars, 200 μm in A & B. (C, D) The iPS cells have alkaline phosphatase activity (C) and express NANOG (D), suggesting stemnss of the human keratinocyte derived iPS cells. Bars, 100 μm in C & D. (E) Teratomas were developed when the iPS cells were inoculated in the testes of SCID mice. Bar, 400 μm. (F–H). The teratomas harbor three germ layer elements, epithelium (F), neural epithelium (G) and cartilage (H), indicating pluripotency of the iPS cells. Bars, 100 μm in F, G, and H.

**Fig 2:**
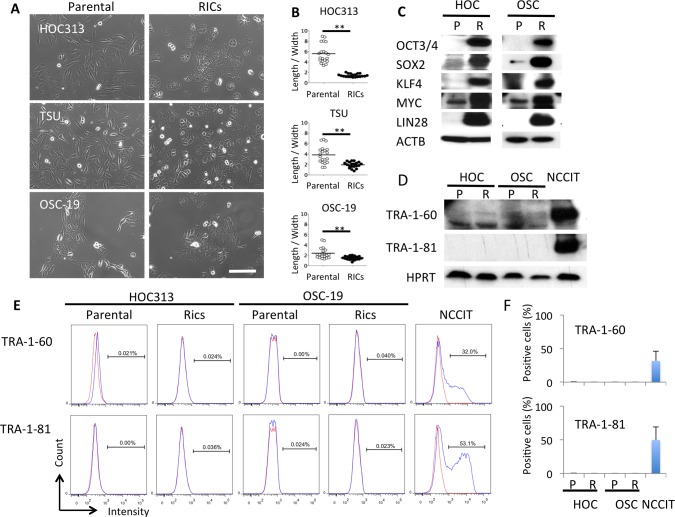
The reprogramming factors introduced SCC cells show epithelial–like morphology but do not express stem cell markers. (A) Parental human SCC cells (left) of HOC313 and TSU show fibroblast-like morphology; however, the reprogramming factors introduced cells (RICs, right panels) exhibit polygonal, epithelial-like morphology. The RICs from OSC-19 cells show more dens cell colony than the parental cells. (Scale bar, 100μm) (B) Cellular morphology is quantified by length to width ratio of each cell from at least 20 cells each from parental SCC cells (open circles) and RICs (closed circles). The ratio of RICs approximates to one, indicating them as epithelial, polygonal shaped cells. ** *P* < 0.01 by Student *t*-test. (C) The introduced reprogramming factors were evaluated by western blot analysis. P, Parental cells. R, RICs. β-Actin (ACTB) was used as loading control. (D-F) Expression of TRA-1-60 and TRA-81 was studied by western blotting (D) and flowcytometry (E, F). The representative data and mean ± SD of three independent experiments of flowcytometry were shown in E and F, respectively. Hypoxanthine-guanine phosphoribosyltransferase (HPRT) was used as loading control in (D). Blue lines; the specific antibodies, red lines; isotype control mouse IgM in (E). NCCIT, a human teratoma cell line, was used as a control in (D-F).

To introduce RFs, we used the piggyBac transposon vector, in which *POU5F1* (*OCT3/4*), *SOX2*, *KLF4*, *cMYC*, and *LIN28* were included [26, 27]. Using this, we could successfully generate iPS cells (Fig 1B) from normal human epidermal keratinocytes (Fig 1A). The iPS cells expressed alkaline phosphatase (Fig 1C) and NANOG (Fig 1D), indicating the stemness. They developed teratomas *in vivo* and showed three germ elements, namely epithelium (Fig 1F), neural epithelium (Fig 1G) and cartilage (Fig 1H). To investigate the impact of RFs on cancer cells *in vitro*, we used three human SCC lines: HOC313, TSU, and OSC-19 cells. Among them, the two cell lines, HOC313 and TSU, had a fibroblast-like spindle shape with increased Snail1 and decreased E-cadherin expression, which indicated that the SCC cells underwent EMT [30]. Using this transposon vector system, the SCC lines were introduced the RFs, which was evaluated by western blotting (Fig 2C). Strikingly, RF-introduced cancer cells (RICs) from all the SCC lines showed a drastic morphological change from spindle to polygonal shape (Fig 2A). Evaluation of cell length/width ratio between parental SCCs and RICs revealed specific reduction in the latter (Fig 2B), suggesting that MET was also induced in SCCs as shown in fibroblast during induction of iPCs by RF [18, 19]. It should be noted that RICs from both cancer lines did not express TRA-1-60 and TRA-1-81, the surface markers for pluripotent cells, whereas a teratoma line NCCIT expressed them by Western blotting (Fig 2D) and flowcytometry (Fig 2E and 2F). This result suggested that RICs did not acquire the pluripotency even after introduction with RFs. To study the impact of MET induced by RFs we used the HOC313 cells and OSC-19 cells for further investigation.

### Mesenchymal-Epithelial Transition Occurs in RICs

**Fig 3:**
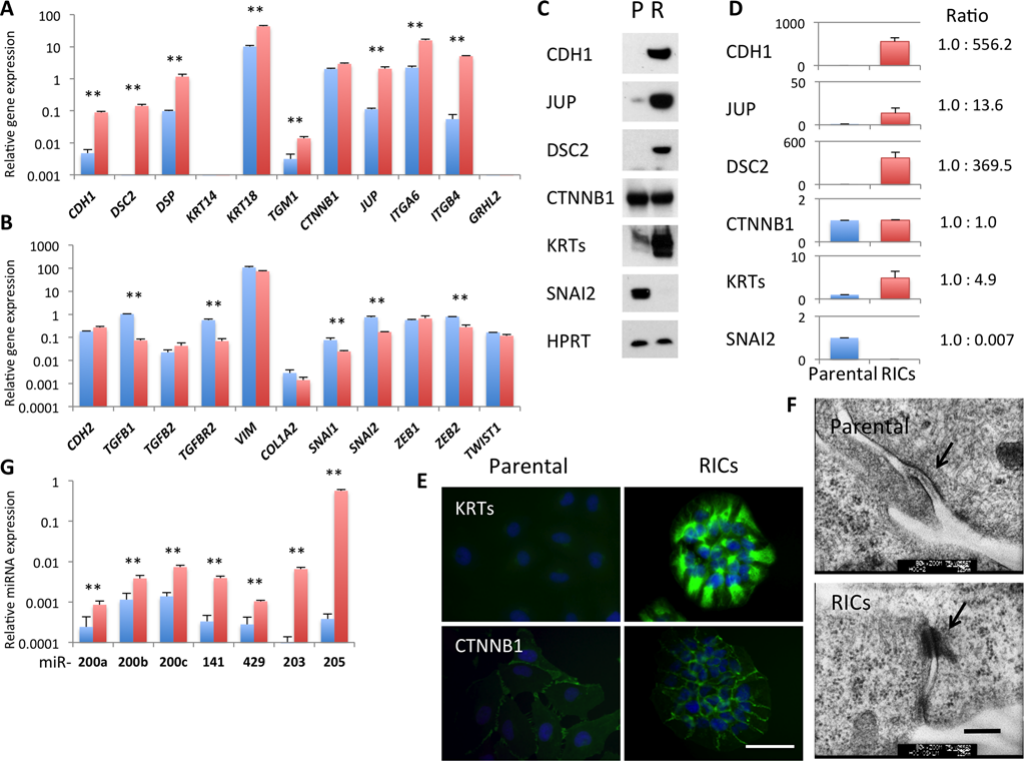
MET in RICs derived from HOC313 cells. (A, B) Alteration of gene expression profile in RICs from HOC313 cells, epithelial marker genes (A) and mesenchymal marker genes (B). The expression of mRNA was normalized with HPRT, and the relative amount of each gene was shown. Blue bars, parental cells. Red bars, RICs. Bars represent mean ± SD from at least three independent experiments. ** *P* < 0.01. Student *t*-test. (C) Western blot analysis. P, Parental cells. R, RICs. (D) The signals of the western analysis were quantified using ImageJ. Signals were normalized with HPRT. Fold change in RICs from parental cells were represented, mean ± SD and mean ratio of three independent experiments. (E) Immunofluorescent staining of parental HOC313 cells and the RICs with anti-pankeratin (KRTs), and β-catenin (CTNNB1) antibodies. (F) Electron micrographs. Arrows indicate desmosome-like structures. Scale bar, 0.2 μm. (G) Expression of miRNAs in RICs (red bars) compared to parental HOC313 cells (blue bars). miRNA was normalized with SNORD61. Bars represent mean ± SD from at least three independent experiments. ** *P* < 0.01 by Student *t*-test.

**Fig 4:**
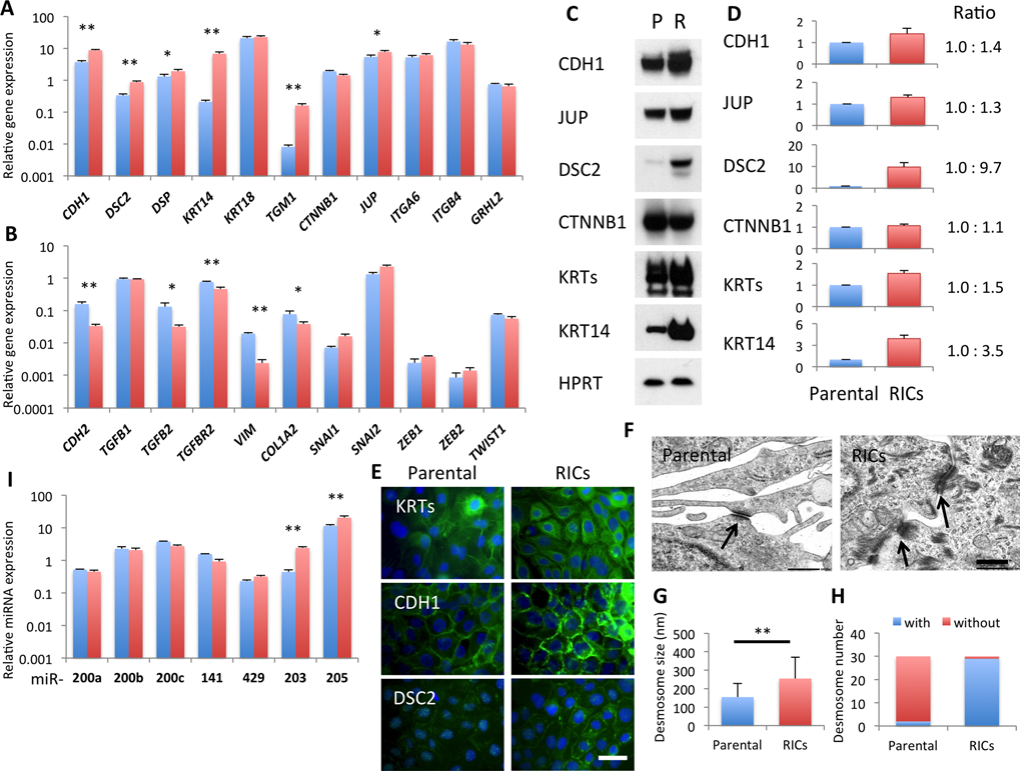
MET in RICs derived from OSC-19 cells. (A, B) Alteration of gene expression profile in RICs from OSC-19 cells, epithelial marker genes (A) and mesenchymal marker genes (B). The expression of mRNA was normalized with HPRT, and the relative amount of each gene was shown. Blue bars, parental cells. Red bars, RICs. Bars represent mean ± SD from at least three independent experiments. * *P* < 0.05, ** *P* < 0.01. Student *t*-test. (C) Western blot analysis. P, Parental cells. R, RICs. (D) The signals of the western analysis were quantified using ImageJ. Signals normalized with HPRT. Fold change in RICs from parental cells were represented, mean ± SD and mean ratio of three independent experiments. (E) Immunofluorescent staining of parental OSC-19 cells and the RICs with anti-pankeratin (KRTs), anti-E-cadherin (CDH1), and anti-desmocollin2 (DSC2) antibodies. (F) Electron micrographs. Arrows indicate desmosome or desmosome-like structures. Scale bar, 0.2 μm. (G) Length of desmosomes or desmosome-like structures on the electron micrographs. Mean ± SD of the 30 objects. ** *P* < 0.01 by Student *t*-test. (H) The desmosomes or desmosome-like structure with tonofilaments were counted among the 30 objects. (I) Expression of miRNAs in RICs (red bars) compared to parental OSC-19 cells (blue bars). miRNA was normalized with SNORD61. Bars represent mean ± SD from at least three independent experiments. ** *P* < 0.01 by Student *t*-test.

We next explored the changes in the expression of epithelial and mesenchymal signature genes in RICs from HOC313 and OSC-19 cells using qRT-PCR. Epithelial signature genes, including *CDH1*, *DSC 2*, *DSP*, *TGM1*, and *JUP* were significantly upregulated in both RICs compared with the respective parental cells (Figs 3A and 4A). Upregulation of *ITGA6* and *ITGB4* in RICs from *HOC313* cells and of *KRT14* in RICs from OSC-19 cells was also observed. However, expression of one of the epithelium-specific transcription factor, grainyhead-like protein 2 homologue (GRHL2) was not changed between parental cells and RICs of both SCC lines.

Conversely, RICs from HOC313 cells showed a significantly reduced expression of mesenchymal signature genes (Fig 3B), including *TGFB1*, *TGFBR2*, *SNAI1*, *SNAI2* and *ZEB2*. OSC-19 RICs showed reduced gene expression of C*DH2*, *TGFB2*, *TGFBR2*, *VIM* and *COL1A2* (Fig 4B). Among the mesenchymal marker genes, *TGFBR2* was commonly downregulated in the RICs from two lines. Western blot analysis detected increased levels of CDH1, JUP (γ-Catenin), DSC2, and KRTs in RICs, which were associated with the epithelial phenotype (Figs 3C and 3D and 4C and 4D). As accordance with q-RT-PCR, loss of SNAI2 and gain of KRT14 were also observed by Western blot in HOC313 RICs and OSC-19 RICs, respectively. Correspondingly, the process of immunostaining revealed that OSC-19 RICs had a marked increase of keratins, CDH1 and DSC2 (Fig 4E). Increased expression of keratins was also observed in HOC313 RICs (Fig 3E). Altered localization of CTNNB1 suggested enhanced cell to cell adhesion in HOC313 RICs (Fig 3E). Furthermore, electron microscopy showed matured desmosome with tonofilaments in HOC313 RICs while parental cells exhibited incomplete desomosome-like structures, where high electron density area was seen on facing two cell membranes (Fig 3F). In OSC-19 RICs, the desmosomes were larger than those in the parental cells and most of them exhibited mature tonofilaments, whereas the parental cells showed immature desmosome with no tonofilaments (Fig 4F and 4H). Collectively, the RICs showed reappearance of desmosomal junctions, which are the major characteristic of well-organized epithelial cells, suggesting MET.

We next examined microRNAs (miRNAs), which reportedly targeted the EMT-inducing molecules. The HOC313 RICs showed increased levels of miR-200 family members (miR-200a, -200b, -200c, -141, and -429), miR-203, and miR-205 (Fig 3G), which could down-regulate the SNAI and ZEB families [15–17]. The OSC-19 RICs showed increased levels of miR-203 and miR-205, although the miR-200 family members were not altered (Fig 4I). This suggested that the introduction of RFs led to expression of EMT-suppressing miRNAs with some different profiles between SCC cells. Conclusively, the RFs induced MET in the human SCC cell lines at the transcriptional and post-transcriptional levels.

### Reprogramming Factors Affect Cell Motility of SCC *in vitro*

**Fig 5:**
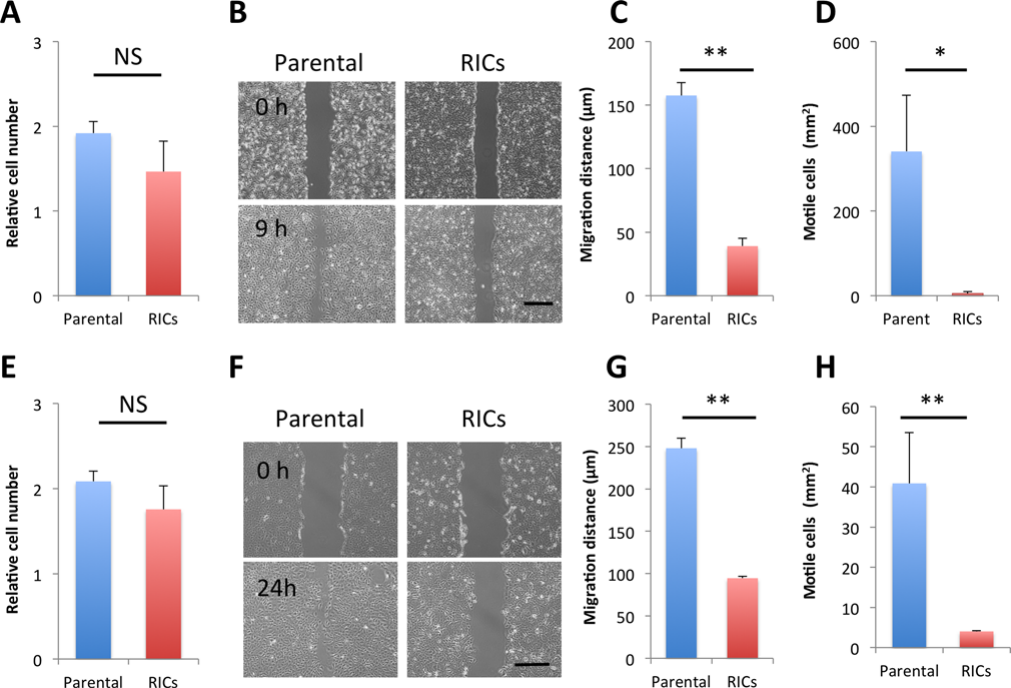
Impaired in vitro migratory and invasive activities in RICs from HOC313 cells and OSC-19 cells. HOC313 parental cells and the RICs (A-D), OSC-19 parental cells and the RICs (E-H). (A, E) Cell proliferation was studied by MTS assay. Relative proliferated cell number for 24 hours was shown. Mean ± SD from three independent experiments. NS, No significant difference. (B, F) *In vitro* wound healing assay revealed that cell migration was markedly impaired in RICs as compared to the parental cells. (Scale bar, 500μm). Representative data are shown from three independent experiments. The times for evaluation are indicated. (C, G) Migrating distance of RICs (red bar) is significantly decreased compared with parental cells (blue bar). Values show mean ± SD. *n* = 3. ** *P* < 0.01 by Student *t*-test. (D, H) Cell invasive activity was reduced in RICs (red bars) as compared to the parental cells (blue bars) using Boyden chambers coated with Matrigel. Values show mean ± SD. *n* = 3. ** *P* < 0.01 by Student *t*-test.

Highly expressed RFs in the RICs (Fig 2C) could have affected the cell proliferation [31], therefore the *in vitro* cell proliferation of RICs was accessed. The MTS assay revealed no significant difference in cell proliferation between the RICs and parental cells (Fig 5A and 5E). Cancer cells under EMT acquired increased cellular motility that led to invasion and metastasis. To assess whether RFs reverse the cell motility of SCC, we used an *in vitro* wound healing assay. The cell migration of RICs from HOC313 and OSC-19 was markedly attenuated as compared to the parental cells (Fig 5B, 5C, 5F and 5G). As compared with the parental cells, the migration ability of RICs was reduced to 25% and 40% in HOC313 RICs and OSC-19 RICs, respectively. Cell invasiveness *in vitro* was evaluated using Boyden chambers in the presence of matrigel coating. The number of RICs, which migrated through the membrane, was significantly smaller than that of the parental cells (Fig 5D and 5H). These results indicate that both cell motility and invasiveness *in vitro* were strikingly impaired in the RICs.

### Reduced *in vivo* Malignancy in RICs

**Fig 6:**
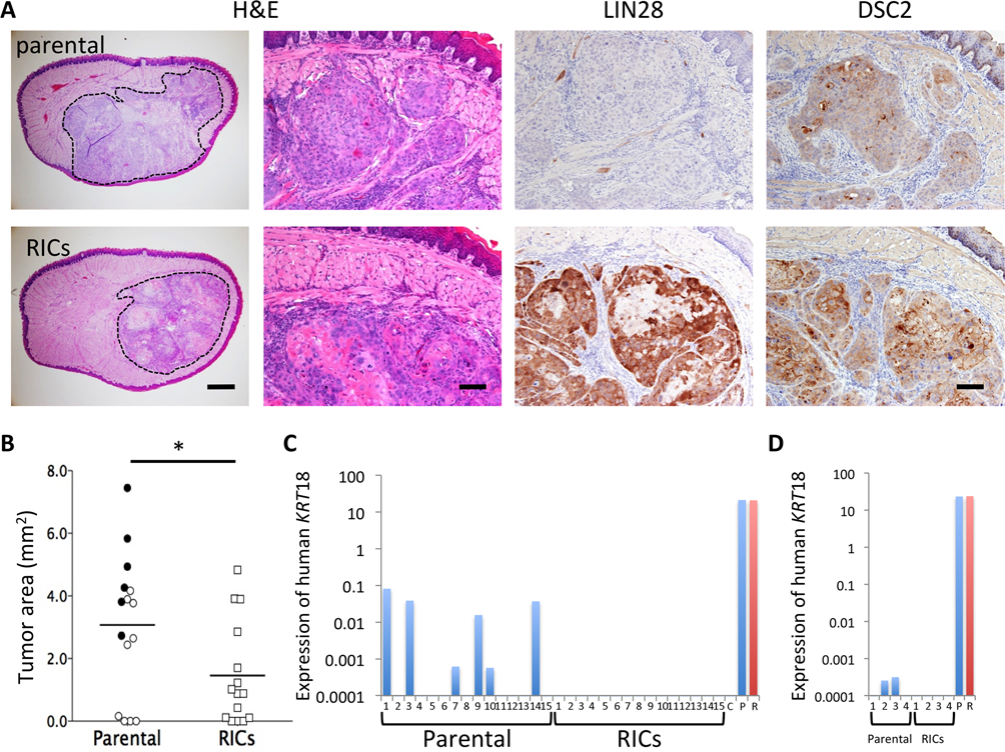
Xenotransplant experiment. (A) Representative images of histology (H&E) and immunohistochemical staining for LIN28 and DSC2 in tumor masses of the tongues 21 days after the inoculation of parental OSC-19 cells and RICs. The tumor area in a tongue tissue was surrounded with a dotted line (left panels of H&E staining). Scale bars, 500 μm and 100 μm in H&E staining and 100 μm in immunostaining. (B) Tumor growth in the tongues of nu/nu mice at day 21 after inoculation of parental cells (circles) and RICs (squares). Closed symbols denote mice exhibiting metastases to lymph nodes. Bars show mean tumor area (mm^2^). *n* = 15 (Cumulative results of three independent experiments. *n* = 5, each group). * *P* < 0.05 by Student *t*-test. (C) Detection of human keratin 18 mRNA from lymph nodes from parental cell-inoculated mice but not from RICs-inoculated mice. Expression of human keratin18 in lymph nodes was detected using qRT-PCR and was normalized with mouse HPRT except for the positive controls, which are normalized with human HPRT. Lymph node metastases occurred in six out of 15 mice inoculated with parental SCC cells in the tongues, in sharp contrast to no metastasis in RIC-inoculated mice. C; control lymph nodes without xenotransplantation, P; parental OSC-19 cells, R; RICs from OSC-19. Expression level of human keratin 18 was almost same for the two cell lines. (D) Detection of human keratin 18 mRNA from lung of nu/nu mice. Specifically amplified signal was detected in 2 of 4 mice in which OSC-19 parental cells were transplanted intravenously. Whereas no signal was detected in OSC-19 RICs transplanted mice.

To study the impact of MET induced by RFs on the attenuation of SCC malignancy, we used the OSC-19 cells, since they were known to metastasize to the cervical lymph nodes when implanted into the lingual margin of nude mice [32]. As demonstrated in the study conducted by Maekawa *et al*. [32], 2.0 × 10^5^ of parental or RIC cells were transplanted into the tongues of nude mice to verify whether RICs showed altered the *in vivo* malignant behavior. Subsequently, the tumor formation in the tongues and metastasis to the draining lymph nodes were evaluated at day 21. Tumors derived from the RICs expressed LIN28, one of RFs (Fig 6A). They also showed higher expression of DSC2 than the parental cells (Fig 6A), indicating that RICs preserved the enhanced epithelial features initially observed *in vitro* (Fig 4A and 4C). The size of tumors formed by RICs (*n* = 15) was significantly smaller than that formed by the parental cells (*n* = 15), indicating the attenuation of *in vivo* growth of the RICs (Fig 6A and 6B). Metastases of the parental OSC-19 cells to the cervical lymph nodes were detected by the presence of human keratin18 expression using RT-PCR and found in 6 out of 15 parental cells inoculated mice, whereas no metastases were found in the RIC-inoculated group (Fig 6C, *P* < 0.01 by Fisher’s exact test). Notably, in mice that were inoculated with parental SCC cells, the sizes of the tumor masses correlated with the probability of metastasis (closed circles in Fig 6B). Indeed, the tumor areas in the tongues of mice harboring metastatic cancer and no metastasis were 4.83 ± 1.65 mm^2^ and 1.90 ± 1.85 mm^2^, respectively (*P* < 0.01 by Student’s t-test). In contrast, the RIC-derived tumors did not metastasize even when they grew as large as tumors of metastasized parental cells (Fig 6B). Furthermore, to investigate metastatic ability of the cells to distant organs, the parental OSC-19 cells and the RICs were transplanted into nude mice via tail vein (2 × 10^5^ cells /mouse). The cell transplanted nude mice lived without any symptom until the experimental end point, day 47 after cell transplant. No tumor growth was seen in thoracic or abdominal organs macroscopically. In addition, no evidence of tumor growth was observed in the lung, liver, spleen, and kidney (data not shown). However, the expression of cytokeratin 18, although it was very low level, was detected from the lung tissues in 2 out of 4 samples of OSC-19 parental cells-inoculated mice, whereas no signal was detected in all the samples from RICs-inoculated mice (Fig 6D). This suggests the presence of lung micrometastasis of OSC-19 parental cells. Thus, the RICs exhibit less malignant potential than the parental cells *in vivo*.

## Discussion

EMT is one of the critical programs of malignant cancer cells that invade the surrounding tissues and metastasize via blood or lymph vessels [1, 3]. Therefore, the induction of the MET program in cancer cells would be relevant in designing a novel anticancer strategy by the restoration of malignant nature of the cancer cells. In the present study, the reprogramming factors were used to induce MET into the cancer cells, which is in line with previous reports that demonstrated MET to occur during the development of iPSCs from fibroblasts [18, 19].

Previous studies demonstrated that reprogrammed malignant cells exhibit an iPSC-like pluripotency to differentiate the cell types into three layers during teratoma formation [20, 21]. Zhang *et al*. [25] have reported that the reprogrammed sarcoma cells had pluripotency to differentiate not only osteocytes and adipocytes but also erythrocytes, which was accompanied with the loss of both proliferation and tumorigenicity. In the study, the authors have showed that the sarcoma cells were dedifferentiated to pre-mesenchymal stem cell state through reprogramming. The RFs via the transposon vector successfully induced iPSC from normal keratinocytes in this study. However, the RICs did not exhibit any iPSC-like characteristics in the current experimental setting and had more differentiated features of epithelial cell lineage as indicated by the alteration of molecular markers and morphology of the tumor cells in vivo, which were distinct from teratomas. Moreover, no previous study had reported a reduced tumorigenicity of partially reprogrammed cancer cells in conjunction with MET induction. As mentioned in previous reports, MET is the crucial and first step of reprogramming caused by combinational action of the RFs, although further appropriate conditions are essential to overcome roadblocks toward pluripotency [33, 34]. We have not applied the culture condition specific for iPS cells when generating the RICs. This might be the reason why they did not demonstrate the pluripotency while MET was induced.

CDH1 is regarded as a gatekeeper of the epithelial state. In the RICs from HOC313 that showed a more drastic change in morphology, the increased expression of *CDH1* and decreased expression of *SNAI1/2* and *ZEB2* were observed. In accordance with this observation, significant up-regulation of miR-200 family, (miR-200a, -200b, -200c, -141, and -429), miR-203, and miR205 was detected in the RICs from HOC313 cells. This result is consistent with the previous study showing that OCT3/4 and SOX2 induce the miR-200 family during iPS generation [35]. Induction of CDH1 was also observed in OSC19 RICs, although the parental OSC-19 cells expressed relatively high level of CDH1 as compared with HOC313 parental cells. Upregulation of CDH1 in OSC-19 RICs may be directly caused by KLF4 [36], since they did not show downregulation of SNAI, ZEB families or up-regulation of miR-200 family. Thus, the sensitivity to RFs of EMT-regulating transcription factors and associated miRNAs varies between SCCs. It has been reported that the down-regulation of miR-203 was observed in some malignant tumor including SCC in head and neck [37–39]. Benaich *et al*. reported that the incidence of lung metastasis was low when SCC cells over-expressed miR-203 [40]. With regard to miR-205, the roles were identified as a tumor suppressor by inhibiting proliferation and invasion, or as an oncogene by facilitating tumor initiation and proliferation in various kinds of tumors [41]. It was reported the miR-205 expression was abundantly found in cutaneous SCC [42], although other studies showed no significant correlation between miR-205 and metastasis in oral SCC [43]. In our study, elevated expression of miR-203 and miR-205 was detected in the two lines of RICs, suggesting that they might act as tumor suppressors in addition to MET inducers by repressing EMT-associated genes.

In the orthotopic xenograft model, the tumor cells derived from human oral SCC OSC-19 cells were inoculated into the tongue. The incidence of lymph node metastasis highlighted the difference between the parental cells and RICs. Although RICs never metastasized even when the primary tumor size was as large as the metastasized parental cells, the tumor growth of the parental cells correlated with the probability of lymph node metastasis. In addition to this, the tumor growth of RICs in the tongues was delayed as compared to the parental cells. In the distant organ metastasis model, the tumor cells were transplanted intravenously. We could detect the expression of human cytokeratin 18 in the lung when inoculated with parental OSC-19 cells but not with RICs. This result clearly indicated attenuated metastatic activity of SCC by introduction of reprogramming factors.

Conversely, some recent studies showed that metastatic breast cancer cells retain the expression of epithelial marker genes such as CDH1, KRT14 and JUP, which could contributed to generation of cancer cell clusters at distant organs of metastatic foci [44–47]. Also, it is known that the process of MET is most relevant for secondary metastases to distal sites. These are quite contradictory to our data, since OSC-19 RICs expressed higher levels of CDH1, JUP, and KRT14. It may be that cancer cells at the primary sites favor the EMT status to invade into the vessels, but once localized at the distant organ they resume the epithelial nature to form cell cluster through MET. Therefore, the RICs failed to metastasize likely because they could not invade into the vessels at the primary lesions. In addition, it may be unlikely that MET promotes secondary metastasis in this experimental setting, since no lung metastasis was formed by intravenous injection of the OSC-19 RICs, in contrast to parental cells forming micrometastases. Alternatively, OSC-19 RICs, even if they reached the lung, might not provide a sufficient condition for the metastatic microenvironment. We could not detect tumor generation from HOC313 cells macroscopically or microscopically, regardless of parental cells and RICs, by inoculation into the subcutaneous or the tongue (data not shown), in contrast to OSC-19, which was derived from oral SCC and has been frequently used in the metastasis model [32]. Other SCC lines than OSC-19 will be required for further investigation to elucidate the association of MET with cancer metastasis, which largely remains to be clarified.

In this study we showed that the MET was induced in SCC cells by the introduction of the reprogramming factors and resulted in the attenuation of cancer malignancy not only *in vitro* but also *in vivo*. The understanding of the process of MET induced by the reprogramming factors in cancer cells will have a considerable impact upon the anticancer strategies in the future.

## Supporting Information

S1 Table Primers used for the study. (PDF)

S2 Table Antibodies used for the study. (PDF)
