# Supplementary material for: Mesenchymal to Epithelial Transition Induced by Reprogramming Factors Attenuates the Malignancy of Cancer Cells
Source: PLoS One. 2016 Jun 3;11(6):e0156904. doi: 10.1371/journal.pone.0156904 (PMC4892607; doi:10.1371/journal.pone.0156904)
Supplement: S1 Table — (PDF) [file pone.0156904.s001.pdf]

S1 Table. Primers used for the study

	Organism	Gene symbol	Sense	Antisense
#1	Human	*CDH1*	GTCCCCCGGTATCTTCCCCGC	CGGGGCTGTGGGGTCAGTATC
#2	Human	*CDH2*	CTCCATGTGCCGGATAGC	CGATTTCACCAGAAGCCTCTAC
#3	Human	*CTTNB1*	AGCGCCGTACGTCCATGGGT	TGTGAAGGGCTCCGGTACAACC
#4	Human	*DSC2*	TCGTCCTGTAGATCGTGAGCAGT	AGGGGCAGTGGAAGTTCTGGAGT
#5	Human	*DSP*	CTGGCCGTGGACCTCTCTTGC	GCCAGGACACCAGGCTCTTCA
#6	Human	*GRHL2*	TGAAAGTCCAGTTTCACCAGAG	TTGAATGGAGGGTCACTGGG
#7	Human	*HPRT*	TGACCTTGATTTATTTTGCATACC	CGAGCAAGACGTTCAGTCCT
#8	Human	*ITGA6*	TTTGAAGATGGGCCTTATGAA	CCCTGAGTCCAAAGAAAAACC
#9	Human	*ITGB4*	CACACTGCCCAGGGACTAC	CAGCAGTCAGGCGAGAGTC
#10	Human	*JUP*	CCTGCTCTGGTCCGCATGCTC	TTGTGCAGCGTGGTGATGGCA
#11	Human	*KRT14*	ATGACCTTGGTGCGGATTT	ATCGAGGACCTGAGGAACAA
#12	Human	*KRT18*	TGATGACACCAATATCACACGA	GGCTTGTAGGCCTTTTACTTCC
#13	Human	*SNAI1*	GCTGCAGGACTCTAATCCAGA	ATCTCCGGAGGTGGGATG
#14	Human	*SNAI2*	TGGTTGCTTCAAGGACACAT	GTTGCAGTGAGGGCAAGAA
#15	Human	*TGFB1*	TGGGATACTGAGACACCCCC	CTCCCGGCAAAAGGTAGGAG
#16	Human	*TGFB2*	CGACGAAGAGTACTACGCCA	GGACTGTCTGGAGCACAAGC
#17	Human	*TGFBR2*	TCTGGCTCAACCACCAGGGCA	TGAGACGGGCCTCTGGGTCG
#18	Human	*TGM1*	CCCCAAGAGACTAGCAGTGG	AGACCAGGCCATTCTTGATG
#19	Human	*TWIST1*	GGCATCACTATGGACTTTCTCTATT	GGCCAGTTTGATCCCAGTATT
#20	Human	*VIM*	GACAATGCGTCTCTGGCACGTCTT	TCCTCCGCCTCCTGCAGGTTCTT
#21	Human	*ZEB1*	GTGTACCAGAGGATGACCTGC	TCCTCCCAGCAGTTCTTAGCA
#22	Human	*ZEB2*	CCCTGGCACAACAACGAGA	GGTCTGGATCGTGGCTTCTG
#23	Mouse	*Hprt*	CACAGGACTAGAACACCTGC	GCTGGTGAAAAGGACCTCT
