# Supplementary material for: Mesenchymal to Epithelial Transition Induced by Reprogramming Factors Attenuates the Malignancy of Cancer Cells
Source: PLoS One. 2016 Jun 3;11(6):e0156904. doi: 10.1371/journal.pone.0156904 (PMC4892607; doi:10.1371/journal.pone.0156904)
Supplement: S2 Table — (PDF) [file pone.0156904.s002.pdf]

S2 Table. Antibodies used for the study

	Symbol	Definition	Company	Product#	*Application
#1	ACTB	β-actin	Sigma	A1978	WB
#2	CDH1	E-cadherin	Cell signaling	3195	WB, IF
#3	CTNNB1	β-catenin	BD	610154	WB, IF
#5	DSC2	Desmocollin 2	Sigma	HPA012615	WB, IF, IHC
#4	JUP	Junction plakoglobin	Novus	NB120-15153	WB
#6	KRT14	Keratin 14	Abcam	ab7800	WB
#7	KRTs	Keratins, wide spectrum	Abcam	ab9377	WB, IF
#8	SNAI2	Snail2, Slug	Abcam	27568	WB
#9	OCT3/4	OCT 3/4, POU5F1	Santa Cruz	sc-5279	WB
#10	SOX2	SOX2	Santa Cruz	sc-20088	WB
#11	KLF4	KLF4	Santa Cruz	sc-20691	WB
#12	MYC	c-MYC	Santa Cruz	sc-764	WB
#13	LIN28	LIN28	Cell signaling	3978	WB, IHC
#14	NANOG	Nanog	Abcam	ab21624	IF
#15	TRA-1-60	TRA-1-60	Millipore	MAB4360	WB, FC
#16	TRA-1-81	TRA-1-81	Millipore	MAB4381	WB, FC
#17	HPRT	hypoxanthine phosphoribosyltransferase	GeneTex	GTX101148	WB

* WB; western blotting
IF; immunofluorescence
IHC; immunohistochemistry
FC; flowcytometry
